# Pain and fatigue are longitudinally and bi-directionally associated with more sedentary time and less standing time in rheumatoid arthritis

**DOI:** 10.1093/rheumatology/keab029

**Published:** 2021-01-26

**Authors:** Ciara M O’Brien, Nikos Ntoumanis, Joan L Duda, George D Kitas, Jet J C S Veldhuijzen van Zanten, George S Metsios, Sally A M Fenton

**Affiliations:** 1 School of Sport, Exercise and Rehabilitation Sciences, University of Birmingham, Birmingham; 2 Department of Rheumatology, Russells Hall Hospital, Dudley Group NHS Foundation Trust, West Midlands; 3 Medical Research Council Versus Arthritis Centre for Musculoskeletal Ageing Research, University of Birmingham, Birmingham, UK; 4 School of Psychology, Faculty of Health Sciences, Curtin University, Perth, Western Australia; 5 Faculty of Education, Health and Wellbeing, University of Wolverhampton, Wolverhampton, UK

**Keywords:** sedentary behaviour, standing, pain, fatigue, rheumatoid arthritis, activPAL

## Abstract

**Objectives:**

The aims of this study were to examine the longitudinal and bi-directional associations of pain and fatigue with sedentary, standing and stepping time in RA.

**Methods:**

People living with RA undertook identical assessments at baseline (T1, *n* = 104) and 6-month follow-up (T2, *n* = 54). Participants completed physical measures (e.g. height, weight, BMI) and routine clinical assessments to characterize RA disease activity (DAS-28). Participants also completed questionnaires to assess physical function (HAQ), pain (McGill Pain Questionnaire) and fatigue (Multidimensional Assessment of Fatigue Scale). Participants’ free-living sedentary, standing and stepping time (min/day) were assessed over 7 days using the activPAL3^µ™^. For the statistical analysis, hierarchical regression analysis was employed to inform the construction of path models, which were subsequently used to examine bi-directional associations of pain and fatigue with sedentary, standing and stepping time. Specifically, where significant associations were observed in longitudinal regression analysis, the bi-directionality of these associations was further investigated via path analysis. For regression analysis, bootstrapping was applied to regression models to account for non-normally distributed data, with significance confirmed using 95% CIs. Where variables were normally distributed, parametric, non-bootstrapped statistics were also examined (significance confirmed via β coefficients, with *P* < 0.05) to ensure all plausible bi-directional associations were examined in path analysis.

**Results:**

Longitudinal bootstrapped regression analysis indicated that from T1 to T2, change in pain, but not fatigue, was positively associated with change in sedentary time. In addition, change in pain and fatigue were negatively related to change in standing time. Longitudinal non-bootstrapped regression analysis demonstrated a significant positive association between change in fatigue with change in sedentary time. Path analysis supported the hypothesized bi-directionality of associations between change in pain and fatigue with change in sedentary time (pain, β = 0.38; fatigue, β = 0.44) and standing time (pain, β = –0.39; fatigue, β = –0.50).

**Conclusion:**

Findings suggest pain and fatigue are longitudinally and bi-directionally associated with sedentary and standing time in RA.

**Rheumatology key messages**

- Pain and fatigue are commonly reported symptoms in people living with RA.
- Pain and fatigue may be a cause and consequence of time spent sedentary in RA.
- Pain and fatigue may be a cause and consequence of time spent standing in RA.

## Introduction

RA is a chronic autoimmune condition characterized by high-grade systemic inflammation, affecting up to 1% of adults worldwide [1]. People with RA commonly experience pain and fatigue [2], and many patients still report these debilitating symptoms when their disease activity is well controlled via pharmacological intervention [3]. Thus, more ‘holistic’, self-management approaches are now endorsed as adjuncts to pharmacological treatment in RA [2, 3].

Physical activity (PA) has been advocated as a non-pharmacological approach to managing RA [4], with prospective and experimental studies consistently reporting that higher levels of moderate-to-vigorous-intensity PA [MVPA, ≥3 metabolic equivalents (METs)] associate with better RA outcomes (e.g. lower disease activity, functional disability, pain and fatigue) [2, 5–7]. However, emerging evidence from cross-sectional studies suggests that high levels of sedentary behaviour (waking activity expending energy ≤1.5 METs, whilst sitting, reclining or lying [8]) are linked to higher disease activity, functional disability and pain among people with RA [9–11].

Pain and fatigue are frequently reported symptoms of RA [2, 5]. Further, the OMERACT initiative recognizes pain and fatigue as core outcome measures in RA research, with only mortality, disablement and irreversible joint damage perceived as more important outcomes to patients [12]. People with RA have reported pain and fatigue as barriers to participation in PA, and reasons for increased engagement in sedentary behaviour [13, 14]. Therefore, the experience of pain and fatigue may initiate a debilitating ‘symptom-activity cycle’, whereby pain and fatigue represent both a cause and a consequence of sedentary behaviour. However, no studies to date have examined the plausible bi-directionality of the associations between pain and fatigue with sedentary time in RA to support the concept of a ‘symptom-activity cycle’. This would confirm scope to intervene and subsequently recommend self-management approaches that target sedentary behaviour change to manage RA-related pain and fatigue. One approach to reducing sedentary time that may be perceived as feasible by people with RA is replacing sedentary behaviour with light-intensity PA (LPA, 1.6–2.9 METs). Indeed, interventions in non-RA populations (e.g. older adults) that target reductions in sedentary time have focused on displacing such behaviours with low-intensity behaviours, such as standing [15].

Therefore, the primary aim of this study was to advance previous cross-sectional studies, and use longitudinal data to explore bi-directional associations between pain and fatigue with daily sedentary time and standing time (a feasible LPA) in RA. A secondary aim was to explore the longitudinal and bi-directional associations between pain and fatigue with an indicator of total PA in RA—daily stepping time.

## Methods

### Participants and recruitment

Patients were recruited from outpatient clinics at Russells Hall Hospital (Dudley, UK). Inclusion criteria were a clinical diagnosis of RA according to the ACR/EULAR classification criteria [16] and aged ≥18 years. Patients who were pregnant, wheelchair users and/or unable to ambulate independently with the use of an assistive device were excluded. All patients provided written informed consent to participate. This study was performed in line with the principles of the Declaration of Helsinki, and was approved by the West Midlands National Health Service Research Ethics Committee (16/WM/0371).

### Protocol

The study methodology is described in detail elsewhere [17]. Briefly, participants visited the hospital at two time points, 6 months apart; time point 1 (T1, baseline) and time point 2 (T2, 6-month follow-up). At T1 and T2, participants visited the hospital twice, separated by 7 days [visit 1 (day 0) and visit 2 (day 7)] to complete the following assessments.

### Measures

#### Visit 1 (day 0)

##### Medical history and demographic information

Participants self-reported their age, sex, ethnicity, marital status, date of diagnosis, existing chronic conditions and current medical treatment.

##### Physical assessments

Height (m), weight (kg), BMI (kg/m^2^) and resting blood pressure (mmHg) were measured in duplicate for each participant.

##### Health assessment questionnaire

The HAQ has been validated in RA, and assesses participants’ physical function by gauging their ability to undertake activities of daily living (ADLs) during the previous 2 weeks [18]. ADLs are categorized into eight sections: ‘dressing and grooming’, ‘rising’, ‘eating’, ‘walking’, ‘hygiene’, ‘reach’, ‘grip’ and ‘activities’. Participants rated how challenging it was to carry out specific tasks associated with each ADL, on a scale from 0 = without any difficulty to 3 = unable to do. Average physical function scores were computed (higher scores indicated poorer physical function: minimum = 0; maximum = 3). The HAQ demonstrated high internal reliability in this study (α = 0.91).

#### Sedentary, standing and stepping time

##### ActivPAL3^µ™^

The activPAL3^µ™^ (PAL Technologies Ltd, Glasgow, UK) is an accelerometer with inclinometer function, which measures habitual behaviour over continuous 24-h periods. This device is considered the gold standard measure of free-living sedentary time [19] and has been validated for measurement of free-living sedentary, standing and stepping time in RA [20].

The activPAL3^µ^ was initialized to record sitting/lying (sedentary), standing and stepping data in 15-s epochs (PAL Connect, PAL Technologies Ltd, Glasgow, UK). The activPAL3^µ^ was fitted to the mid-anterior position of the participants’ right thigh by the researcher, using an adhesive, waterproof dressing [21]. Participants were asked to wear the activPAL3^µ^ for 24 h/day for the subsequent 7 days, and record any removal/replacement of the device in a wear time logbook.

Participants’ data were downloaded from the activPAL3^µ^ and exported to Microsoft Excel for analysis. The researcher manually examined activPAL3^µ^ data to determine periods of sleep, and these were subsequently removed from sedentary time estimates. To be included in statistical analysis, participants must have worn the activPAL3^µ^ for ≥10 h/day on ≥4 days of the week (including one or more weekend day) [21] at T1 and T2.

For participants with valid activPAL3^µ^ data, average daily waking time (min/day) and average daily percentage of waking time (%) spent sedentary, standing and stepping were calculated and used in statistical analysis {e.g. sedentary time/day (%) = [sedentary time (min/day)/total wear time (min/day)] × 100}.

#### Visit 2 (day 7)

##### Disease activity score-28

The DAS-28 assessed RA disease activity [22]. The number of swollen and tender joints in 28 synovial joints (hands, wrists, elbows, shoulders and knees), ESR (mm/h) and self-reported degree of overall health (100 mm visual analogue scale: 0 = very good to 100 = very poor) was used to compute participants’ DAS-28 using a validated clinical calculator (https://www.das-score.nl/das28/DAScalculators/dasculators.html).

##### McGill pain questionnaire

The McGill Pain Questionnaire (MPQ) is a multidimensional instrument used to measure pain, and has been validated in RA [23, 24]. The MPQ includes 15 items, encompassing sensory (11 items, e.g. ‘sharp’) and affective (4 items, e.g. ‘tiring-exhausting’) dimensions of pain. Participants rated to what extent they identified with each descriptor (0 = none; 1 = mild; 2 = moderate; 3 = severe) over the previous 7 days. Responses were summed to compute a total pain score (higher scores indicated poorer pain outcomes: minimum = 0; maximum = 45). In this study, high internal reliability was demonstrated for the MPQ (α = 0.93).

##### Multidimensional assessment of fatigue scale

The Multidimensional Assessment of Fatigue Scale (MAF) is a 15-item measure of global fatigue, developed and validated in RA [25]. This scale has been extensively employed in previous studies of fatigue in RA [26, 27]. The MAF required participants to rate their degree of fatigue, and to what extent fatigue interfered with their ability to carry out ADLs (e.g. ‘household chores’) over the previous 7 days on a scale from 1 = not at all to 10 = a great deal. A global fatigue index was calculated (higher scores related to poorer fatigue outcomes: minimum = 0; maximum = 50). In this study, the MAF showed high internal reliability (α = 0.98).

### Statistical analysis

Statistical analysis were conducted using SPSS and AMOS software (v.24) (IBM Corp.). Descriptive statistics were computed for all variables at T1 and T2, along with change scores (change score = T2 – T1). Kolmogorov-Smirnov tests were conducted to check normality of the data. Bias-corrected and accelerated bootstrapping [28] was employed in all regression and path analysis to account for any non-normally distributed data. Bootstrapping is a non-parametric resampling procedure that does not impose the assumption of normal distribution and simulates obtaining data from a large sample [29, 30].

Bivariate correlation and hierarchical regression analysis examined longitudinal (change from T1 to T2) associations between pain and fatigue with activPAL3^µ^-assessed sedentary, standing and stepping time. Regression models were constructed to align with a ‘clinical perspective’, in which RA-related pain and fatigue is first hypothesized to influence habitual behaviours (e.g. sedentary time) [31]. Therefore, pain and fatigue were entered (separately) as independent variables, and activPAL3^µ^-assessed sedentary, standing and stepping time (%) were entered (separately) as dependent variables, in regression analysis. Regression models were adjusted for activPAL3^µ^ wear time (model 1), then further adjusted for age and sex (model 2).

The direction and statistical significance of associations explored in regression analysis were determined by examining bootstrapped unstandardized coefficients (*B*) and 95% CIs. Significant relationships were inferred where bootstrapped 95% CIs did not cross zero. Non-bootstrapped standardized coefficients (β) were also computed to aid interpretation of the strength of observed relationships (small = 0.10; medium = 0.30; large = 0.50) [32]. *R*^2^ values represented the unique variance in activPAL3^µ^-assessed sedentary, standing or stepping time, explained by pain or fatigue.

Following regression analysis, path analysis was conducted to examine the bi-directional relationships between pain and fatigue with activPAL3^µ^-assessed behaviours. Primarily, where pain or fatigue were significantly ‘predicting’ activPAL3^µ^-assessed sedentary, standing or stepping time in longitudinal bootstrapped regressions, the plausible bi-directionality of these associations was explored in path analysis. Where bootstrapped regression analysis revealed non-significant associations between pain or fatigue with activPAL3^µ^-assessed behaviours, but variables included in that specific analysis were normally distributed (did not violate assumptions required to conduct parametric statistical tests), significant non-bootstrapped standardized coefficients (β) prompted further exploration of a bi-directional association in path analysis.

**Fig. 1:**
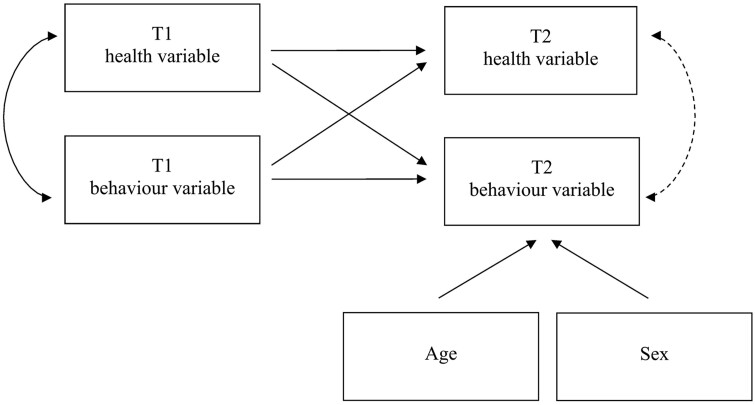
Path model used to test for bi-directional associations Path models were adjusted for age and sex, but are not shown in Fig. 2 for ease of interpretation. Arrow: path specified between variables; Arrow (dotted line): bi-directional association tested; T1: time point 1; T2: time point 2; health variable: self-reported pain or fatigue; behaviour variable: activPAL3^µ™^-assessed sedentary, standing or stepping time.

Fig. 1 illustrates the model employed to investigate bi-directional associations between pain and fatigue with activPAL3^µ^-assessed behaviours. The ‘T2 health variable’ (pain or fatigue) and ‘T2 behaviour variable’ (sedentary, standing or stepping time) represent change in the variable from T1 to T2. Since T1 variables are controlled for, T2 variables in the model represent ‘change scores’. By correlating the T2 health and behaviour variables, whether the changes in these variables are significantly associated with one another can be tested—a significant association supports the presence of bi-directionality. Path models were adjusted for age and sex.

Significant bi-directional associations were inferred where bootstrapped 95% CIs, representing the association between T2 health and behaviour variables, did not cross zero. Standardized coefficients (β) were also reported to indicate the effect size of the associations. Model fit indices were used to confirm the stability of the parameter estimates. Specifically, model fit was assessed using the *χ*^2^ statistic (*χ*^2^), comparative fit index (CFI), Tucker Lewis Index and root mean square error of approximation (RMSEA, 90% CIs). ‘Excellent’ model fit criteria were: non-significant *χ*^2^ (*P* > 0.05), CFI and Tucker Lewis Index values ≥0.95, and RMSEA <0.06 with 90% CIs (lower boundary) containing 0. ‘Good’ model fit criteria were: non-significant *χ*^2^ (*P* > 0.05), CFI and Tucker Lewis Index values ≥0.90, and RMSEA <0.08 with 90% CIs (lower boundary) <0.05 [33, 34].

## Results

A total of *n *=* *104 participants were recruited at baseline (T1), and *n *=* *54 participants (52% of the sample from T1) undertook assessments 6 months later (T2). The research team were not able to follow-up with the remaining *n *=* *50 participants to complete T2 assessments due to time and study funding constraints. At T1 and T2, 98% of participants who completed the protocol at these time points provided valid activPAL3^µ^ data (T1, *n *=* *102 out of 104 participants; T2, *n *=* *53 out of 54 participants). The final sample available for longitudinal analysis was therefore *n *=* *53. Descriptive statistics for the sample are reported in Table 1.

**Table 1:** Descriptive statistics for the sample at T1 and T2, and change from T1 to T2

	*n*	T1	*n*	T2	*n*	Change
Age (years)	102	58.3 (12.3)	53	58.9 (12.2)	–	–
Sex (% female)	72	71	37	70	–	–
Ethnicity (% Caucasian)	97	95	51	96	–	–
Marital status (% married)	66	65	38	70	–	–
RA disease						
RA duration (years)	102	10.4 (10.5)	53	9.0 (8.1)	–	–
DAS-28	102	4.0 (1.5)	53	4.0 (1.5)	–	–
Phy*sic*al function (HAQ)	102	1.2 (0.8)	53	1.0 (0.8)	–	–
DMARDs (% on DMARDs)	92	90	46	87	–	–
Anti-TNF (% on anti-TNF)	15	14	11	20	–	–
NSAIDs (% on NSAIDs)	19	18	11	20	–	–
Physical health						
Height (m)	102	1.7 (0.1)	53	1.7 (0.1)	–	–
Weight (kg)	102	80.0 (20.3)	53	81.7 (22.0)	–	–
BMI (kg/m^2^)	102	29.1 (6.1)	53	29.7 (6.6)	–	–
Systolic BP (mmHg)	102	129 (15)	53	132 (13)	–	–
Diastolic BP (mmHg)	102	77 (9)	53	77 (8)	–	–
RA outcomes						
Pain (MPQ)	102	12.8 (11.0)	53	13.4 (11.0)	53	–0.7 (10.0)
Fatigue (MAF)	102	24.8 (13.2)	53	23.6 (13.2)	53	–1.5 (8.7)
ActivPAL3^µ™^ data						
Valid wear time (min/day)	102	913.0 (56.7)	53	941.3 (60.4)	53	20.5 (54.2)
Sedentary time (min/day)	102	546.1 (116.6)	53	574.8 (98.8)	53	37.9 (65.3)
Standing time (min/day)	102	267.5 (101.0)	53	266.6 (92.7)	53	–13.1 (59.9)
Stepping time (min/day)	102	99.4 (37.4)	53	99.9 (40.3)	53	–4.3 (19.8)
Sedentary time (%/day)	102	60.0 (12.9)	53	61.4 (11.6)	53	2.8 (6.8)
Standing time (%/day)	102	29.2 (10.5)	53	28.1 (8.9)	53	–2.1 (5.9)
Stepping time (%/day)	102	10.9 (4.0)	53	10.5 (4.0)	53	–0.7 (2.2)

Values are percentages (%) or mean (s.d.). *n*: number of participants; T1: time point 1; T2: time point 2; –:not applicable; BP: blood pressure; MPQ: McGill Pain Questionnaire; MAF: Multidimensional Assessment of Fatigue Scale.

### Bivariate correlation analysis

Table 2 reports results from longitudinal bivariate correlations. Change in pain and fatigue were significantly negatively linked with change in standing time, but were not related to change in sedentary or stepping time.

**Table 2:** Bivariate Pearson’s correlations between pain and fatigue with activPAL3^µ™^-assessed sedentary, standing and stepping time (longitudinal)

	1	2	3	4
1 Pain				
2 Fatigue	0.57*			
3 Sedentary time	0.26	0.27		
4 Standing time	–0.27*	–0.29*	–0.95^**^	
5 Stepping time	–0.07	–0.05	–0.55*	0.27

* *P* < 0.05; **P < 0.01. Bivariate Pearson’s correlations were adjusted for activPAL3^µ™^ wear time. Sedentary, standing and stepping time were calculated as percentages of activPAL3^µ™^ wear time for use in bivariate Pearson’s correlations.

### Longitudinal regression analysis

Table 3 displays results from longitudinal regression analysis. After adjusting for age and sex, bootstrapped regression analysis revealed that change in pain was significantly positively associated with change in sedentary time, predicting 7% of the variance in this behaviour. Change in fatigue was not significantly related to change in sedentary time. Change in pain and fatigue were significantly negatively associated with change in standing time, accounting for 8% and 9% of the variance in this behaviour, respectively. No significant relationships were demonstrated for change in pain or fatigue with change in stepping time. All significant associations were of a small to medium effect size [32].

**Table 3:** Linear regressions between pain and fatigue with activPAL3^µ™^-assessed sedentary, standing and stepping time (longitudinal)

	Sedentary time				Standing time				Stepping time			
	*B*	95% CIs	*R* ^2^	β	*B*	95% Cis	*R* ^2^	β	*B*	95% CIs	*R* ^2^	β
1 Pain	0.18*	0.01, 0.32	0.07	0.26^a^	–0.16*	–0.31, –0.02	0.08	–0.27*	–0.02	–0.06, 0.05	0.01	–0.07
2 Pain	0.19*	0.01, 0.35		0.28^a^	–0.18*	–0.34, –0.02		–0.30*	–0.01	–0.07, 0.06		–0.06
Age	0.65	–2.89, 4.46	0.00	0.05	0.12	–2.73, 2.78	0.00	0.01	–0.75	–1.96, 0.49	0.03	–0.18
Sex	–1.54	–5.93, 2.08	0.01	–0.11	2.06	–1.61, 5.97	0.03	0.16	–0.52	–1.70, 0.69	0.01	–0.11
1 Fatigue	0.21	–0.00, 0.51	0.07	0.27	–0.20*	–0.42, –0.03	0.09	–0.29*	–0.01	–0.08, 0.05	0.00	–0.05
2 Fatigue	0.22	–0.01, 0.52		0.29*^a^	–0.22*	–0.45, –0.03		–0.32*	–0.01	–0.08, 0.06		–0.02
Age	0.42	–3.42, 4.22	0.00	0.03	0.33	–2.65, 3.00	0.00	0.03	–0.73	–1.87, 0.46	0.03	–0.17
Sex	–1.68	–6.53, 2.72	0.01	–0.11	2.23	–0.99, 5.13	0.03	0.17	–0.55	–1.75, 0.47	0.01	–0.12

* 95% CIs (lower, upper) do not cross zero or *P* < 0.05. Model ‘1’ adjusted for activPAL3^µ™^ wear time. Model ‘2’ adjusted for activPAL3^µ™^ wear time, age and sex. Sedentary, standing and stepping time were calculated as percentages of activPAL3^µ™^ wear time for use in regression analysis. ^a^Significant/non-significant result for non-bootstrapped standardized coefficient was different from bootstrapped unstandardized coefficient. *B*: unstandardized coefficient (from bootstrapped data); β: standardized beta coefficient; *R*^2^: variance explained in the dependent variable (sedentary, standing or stepping time) by the independent variable (pain or fatigue).

Whilst bootstrapped analysis indicated no significant association between change in fatigue with change in sedentary time, non-bootstrapped analysis showed a significant positive association between these variables (β = 0.29, *P* < 0.05). Given that fatigue and sedentary time were normally distributed variables, the plausible bi-directionality of this association was examined.

### Bi-directional path analysis

Informed by regression analysis, four path models were tested to examine bi-directional relationships between change in pain and fatigue with change in sedentary and standing time in RA.

**Fig. 2:**
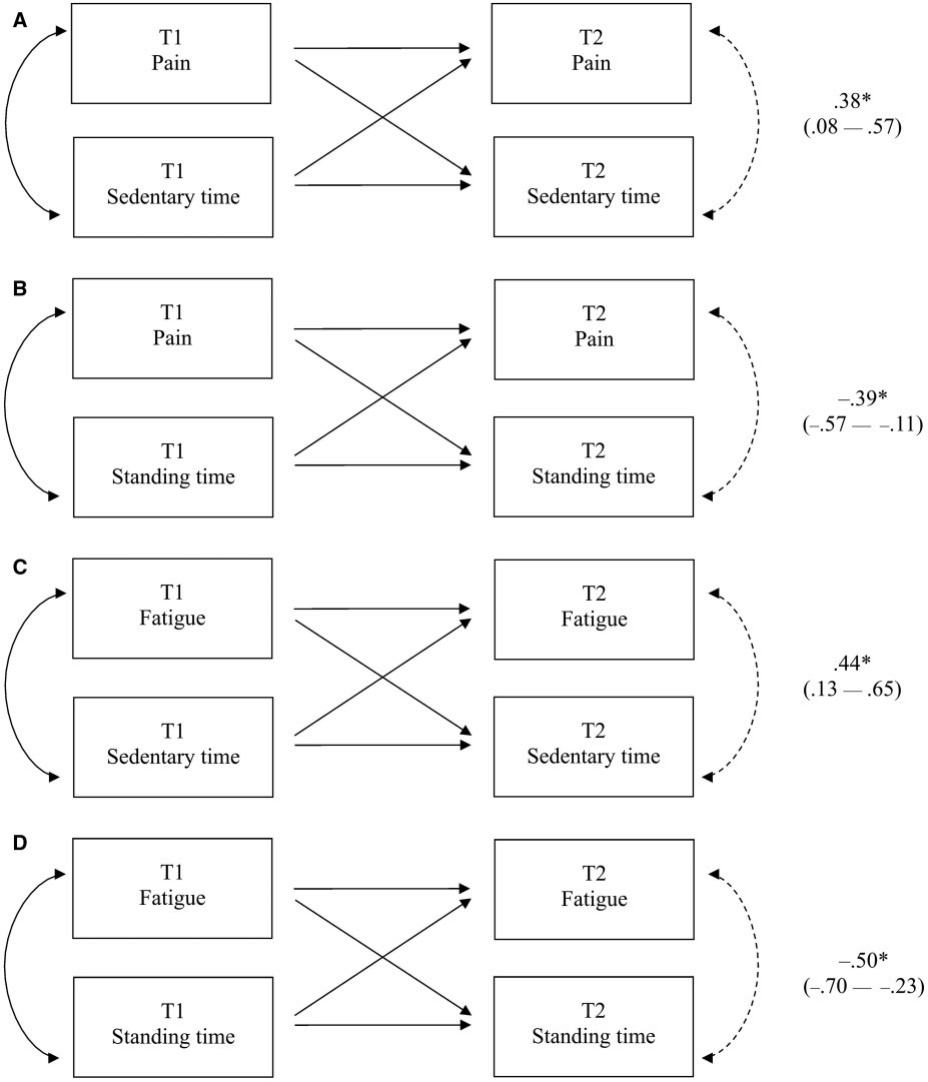
Path analysis revealing bi-directional associations Path analysis revealing bi-directional associations between: pain with sedentary time (**A**); pain with standing time (**B**); fatigue with sedentary time (**C**); fatigue with standing time (**D**). *95% CIs do not cross zero. Standardized coefficients (β) and 95% CIs (lower – upper) are reported. Sedentary and standing time were calculated as percentages of activPAL3^µ™^ wear time for use in bi-directional path analysis. All path models were adjusted for age and sex, but are not shown in this figure for ease of interpretation. Arrow: path specified between variables; Arrow (dotted line): bi-directional association tested; T1: time point 1; T2: time point 2.

Bi-directional path models are displayed in Fig. 2. Pain and fatigue showed significant positive bi-directional associations with sedentary time, and significant negative bi-directional relationships with standing time. All models demonstrated excellent fit to the data (Table 4).

**Table 4:** Model fit: Bi-directional associations between pain and fatigue with activPAL3^µ™^-assessed sedentary and standing time (longitudinal)

Health variable	Behaviour variable	*χ* ^2^	CFI	Tucker Lewis Index	RMSEA	90% CIs	PCLOSE	Model fit
Pain	Sedentary time	4.19, *P* = 0.76	1.00	1.06	0.00	0.00, 0.12	0.81	Excellent
Pain	Standing time	4.72, *P* = 0.69	1.00	1.06	0.00	0.00, 0.13	0.76	Excellent
Fatigue	Sedentary time	6.91, *P* = 0.44	1.00	1.00	0.00	0.00, 0.17	0.53	Excellent
Fatigue	Standing time	6.82, *P* = 0.45	1.00	1.00	0.00	0.00, 0.17	0.54	Excellent

For *χ*^2^, degrees of freedom were 7. RMSEA encompasses 90% CIs (lower, upper) and PCLOSE for assessing model fit. *χ*^2^: *χ*^2^ statistic; CFI: comparative fit index; RMSEA: root mean square error of approximation; PCLOSE: closeness of fit.

## Discussion

This is the first study to examine the longitudinal relationships between change in pain and fatigue with change in habitual sedentary, standing and stepping time in people with RA. Results from bootstrapped regression analysis revealed that change in pain, but not fatigue, was significantly positively associated with change in sedentary time, and change in pain and fatigue were significantly negatively related to change in standing time, in RA. When considering the normal distribution of fatigue and sedentary time, non-bootstrapped regression analysis showed a significant positive relationship between change in fatigue with change in sedentary time. Subsequent path analysis demonstrated that the associations between change in both pain and fatigue with sedentary and standing time were bi-directional. That is, pain and fatigue may represent both determinants and consequences of sedentary and standing time in this patient group.

A recent cross-sectional study conducted by O’Leary *et al.* [11] provided preliminary evidence regarding the relationships between pain and fatigue with sedentary and standing time in RA. Specifically, O’Leary and colleagues reported significant positive associations between indicators of pain (pain intensity, number of painful joints, and presence of foot and/or ankle pain) with activPAL4™-assessed sedentary time in RA, but did not observe significant associations between fatigue with activPAL4-assessed sedentary time. Interestingly, these authors also reported significant negative associations between the intensity of pain and fatigue with activPAL4-assessed standing time.

The current study extends the cross-sectional work of O’Leary *et al.* by employing a longitudinal design and providing evidence for the bi-directionality of these associations. In addition, pain and fatigue are experienced differently between individuals. Thus, when measuring RA-related pain and fatigue, it is more insightful to capture their multidimensional nature than just their intensity [35–37]. Adding to the findings of O’Leary and colleagues, the current study goes beyond examining pain intensity to capture multidimensional measures of pain (sensory and affective pain, resulting in a total pain score) and global fatigue (degree, severity, distress, timing and impact on ADLs, resulting in a global fatigue index) via employment of the MPQ and MAF, respectively [25, 35, 36]. Indeed, the MPQ and MAF are widely used multidimensional measures of pain and fatigue, and have been validated for use in RA [23–27].

This study provides novel data to confirm the presence of bi-directional associations between pain and fatigue with sedentary and standing time in RA. That is, high levels of sedentary time and low levels of PA may not only represent consequences of RA disease symptomology, but may also act as contributors to variability in RA outcomes [13, 14]. As such, targeting reductions in sedentary time in people with RA, concurrent to increasing standing time, may represent one avenue by which these important disease outcomes could be improved. Indeed, the strong inverse correlation demonstrated between sedentary and standing time (β = –0.95) suggests that interventions which target replacing sedentary time with standing may offer a feasible approach to ameliorate the possible negative consequences of sedentarity in RA. Moreover, the standardized path coefficients representative of the bi-directional associations between pain and fatigue with sedentary and standing time were similar [e.g. pain with sedentary (β = 0.38) and standing (β = –0.39) time]. Together, these findings substantiate the potential health impacts for people with RA that may result from replacing sedentary time with lower-intensity PA behaviours, such as standing (‘sit less, stand more’). This links well to very recent national PA and sedentary behaviour guidelines for adults and older adults, which strongly recommend limiting sedentary time by replacing it with PA of any intensity (including LPA) for health benefits [38]. Experimental studies, however, are required to confirm the causality of associations emerging from this observational study.

The current longitudinal study revealed that pain and fatigue could be facilitators of sedentary behaviour and barriers to LPA (standing) in RA, supporting previous work in this field [13, 14]. These disease factors may represent less salient determinants of sedentary behaviour and standing than MVPA (the traditional focus of interventions in this patient group). Still, evidence for bi-directional associations between pain and fatigue with sedentary and standing time in this study highlight the importance of considering these factors in the development of sedentary behaviour change interventions in people with RA. Recently, EULAR have highlighted the need for more high quality RA studies investigating the important relationships between sedentary behaviour and LPA with health indicators in this population, to inform the design of such interventions [4]. Moreover, the presence of bi-directional associations underlines the importance of identifying other factors that influence sedentary and standing time in RA, over and above pain and fatigue, to design interventions that can inspire behaviour change even when individuals experience pain and fatigue. Psychological theories may offer useful frameworks for identifying modifiable determinants that can be targeted by intervention (e.g. motivation to reduce sedentary behaviour) [39].

In discerning the clinical relevance of these findings from an intervention standpoint, results suggest a decrease in sedentary time of ∼33 min/day may lead to a decrease in both MPQ score (pain) and MAF score (fatigue) by 2 on each scale. For example, for RA patients who initially report experiencing ‘severe’ pain (rated as 3 on the MPQ) and fatigue ‘every day’ (rated as 4 on the MAF), reducing sedentary time (or increasing standing time) by approximately half an hour per day may equate to a change whereby these patients report ‘mild’ pain (rated as 1 on the MPQ) and fatigue ‘occasionally, but not most days’ (rated as 2 on the MAF).

This study’s findings demonstrated that pain and fatigue were not significantly related to stepping time in RA. ActivPAL3^µ^-assessed stepping time is typically considered an indicator of total PA, as few studies have examined the accuracy of the activPAL3^µ^ for assessing the intensity of free-living PA [40]. Recent evidence suggests that greater levels of total PA (inclusive of LPA and MVPA) and lower levels of sedentary time are linked to lesser risk of premature all-cause mortality in middle-aged and older adults [41]. Results from the current study suggest that specific RA disease outcomes, such as pain and fatigue, may hold different associations with total PA. Specifically, it may be that changes in behaviours lower down on the ‘activity continuum’ (sedentary behaviour and LPA) are more important for inducing changes in RA-related pain and fatigue in this patient group. Research that further examines the role of intensity *vs* overall volume of PA for a broad range of outcomes in RA, including pain and fatigue, is therefore required. Paramount to this research is that measures employed to assess PA intensity and volume have been validated specifically for use in this population, or are based on standardized metrics that do not require arbitrary decisions in accelerometer data processing (e.g. average acceleration), which can impact conclusions [42].

Limitations to this study should be acknowledged. Some participants recruited at T1 in this longitudinal study were not followed up at T2 due an end to study funding. However, no significant differences were found between participants included at T2 and those lost between T1 and T2 for all measured variables. Furthermore, bootstrapping was employed in statistical analysis which is suggested to provide more accurate parameter estimates in studies with smaller samples [29]. Nevertheless, the sample size meant it was not possible to test more sophisticated statistical models, including more complex structural equation models and bi-directional models exploring associations between pain and fatigue with sedentary and standing time synonymously. In addition, the sample consisted of mostly female participants with moderate disease activity and severity, limiting the generalizability of findings to males with RA and those with more/less active disease. However, it should be noted that a higher proportion of females is representative of the RA population [1], and participants’ disease activity (DAS-28), disease severity (HAQ), and self-reported pain (MPQ) and fatigue (MAF) in this study closely reflect descriptive data from previous studies in this patient group [27, 43–45].

## Conclusion

This study revealed longitudinal associations between change in pain and fatigue with change in habitual sedentary and standing time in people with RA. Results suggest that variability in pain and fatigue may represent both determinants and consequences of sedentary and standing time in this patient group. Future research should employ experimental study designs to test whether replacing sedentary time with standing improves pain and fatigue in RA. Additionally, investigation into other factors that influence sedentary, standing and stepping time in RA, over and above pain and fatigue, is warranted—particularly, the modifiable determinants of these behaviours which could be targeted in sedentary behaviour change interventions.
